# Polarity in Ciliate Models: From Cilia to Cell Architecture

**DOI:** 10.3389/fcell.2019.00240

**Published:** 2019-10-18

**Authors:** Helena Soares, Bruno Carmona, Sofia Nolasco, Luís Viseu Melo

**Affiliations:** ^1^Centro de Química e Bioquímica/Centro de Química Estrutural, Faculdade de Ciências, Universidade de Lisboa, Lisbon, Portugal; ^2^Escola Superior de Tecnologia da Saúde de Lisboa, Instituto Politécnico de Lisboa, Lisbon, Portugal; ^3^CIISA-Centro de Investigação Interdisciplinar em Sanidade Animal, Faculdade de Medicina Veterinária, Universidade de Lisboa, Lisbon, Portugal; ^4^Physics Department and CEFEMA, Instituto Superior Técnico, Universidade de Lisboa, Lisbon, Portugal

**Keywords:** cell polarity, cell patterning, ciliates cortex, signaling, cilia, basal bodies, AFM

## Abstract

*Tetrahymena* and *Paramecium* are highly differentiated unicellular organisms with elaborated cortical patterns showing a regular arrangement of hundreds to thousands of basal bodies in longitudinal rows that extend from the anterior to the posterior region of the cell. Thus both ciliates exhibit a permanent antero–posterior axis and left–right asymmetry. This cell polarity is reflected in the direction of the structures nucleated around each basal body such as the ciliary rootlets. Studies in these ciliates showed that basal bodies assemble two types of cilia, the cortical cilia and the cilia of the oral apparatus, a complex structure specialized in food capture. These two cilia types display structural differences at their tip domain. Basal bodies possessing distinct compositions creating specialized landmarks are also present. Cilia might be expected to express and transmit polarities throughout signaling pathways given their recognized role in signal transduction. This review will focus on how local polarities in basal bodies/cilia are regulated and transmitted through cell division in order to maintain the global polarity and shape of these cells and locally constrain the interpretation of signals by different cilia. We will also discuss ciliates as excellent biological models to study development and morphogenetic mechanisms and their relationship with cilia diversity and function in metazoans.

## Introduction: The Complex Architecture of a Single Cell Organism

Ciliates are a large and diverse group of unicellular eukaryotes that present a variety of shapes and sizes. They are ecologically important and virtually disperse over all freshwater, marine, and terrestrial environments, maintaining symbiotic relationships with a vast microbiome (Nowack and Melkonian, 2010). Ciliates present a nuclear dimorphism characterized by the separation of the germ-line from the somatic-line. The polyploid macronucleus (the somatic nucleus) assures the vegetative growth of cells, and the diploid micronucleus (the gametic nucleus) is only transcriptionally active during sexual conjugation (Prescott, 1994).

These organisms are permanently polarized cells characterized by possessing a high diversity of microtubule (MT) structures that guarantee cell survival. For example, in *Tetrahymena thermophila* at least 18 different functional MTs are involved in different functions such as feeding, cell division, sexual conjugation, cell motility, and cell architecture (for review Frankel, 2000; Gaertig, 2000; Wloga and Gaertig, 2010). This structural and functional diversity in a unique cell parallels to what is observed in complete metazoan organisms (detailed revision Frankel, 2000; Gaertig, 2000; Wloga and Frankel, 2012).

Cilia and basal bodies (BB) are prominent MTs based complex organelles of the ciliate cell. A typical *Tetrahymena* cell (40 to 50 μm long) will present about 750 BBs distributed through 18–21 antero-posterior rows and ∼150 BBs at the oral apparatus (OA) (for review Pearson and Winey, 2009). In the larger cell of *Paramecium* (∼120 μm long) 4,000 BBs localize in 70 longitudinal rows being 1,000 BBs at the OA (for review Pearson and Winey, 2009).

**FIGURE 1:**
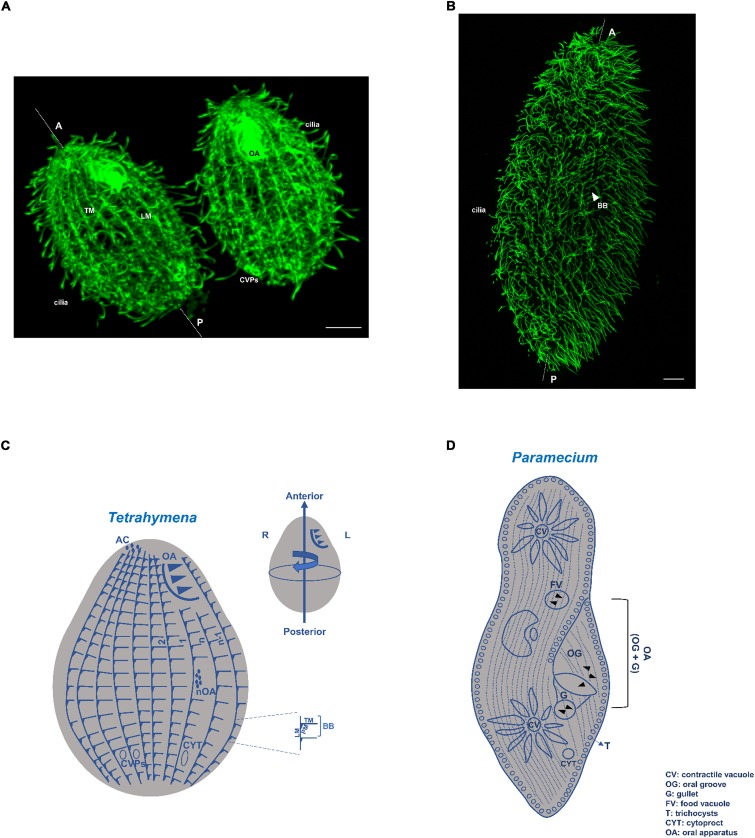
*Tetrahymena* and *Paramecium* cortical structures based on microtubules. **(A)** Immunofluorescence microscopy using an antibody against α-tubulin (12G10 antibody) of a *T. thermophila* exponentially growing cell. Scale bar = 10 μm. **(B)** Immunofluorescence microscopy using an antibody against glutamylated tubulin (PolyE antibody) of a *P. tetraurelia* exponentially growing cell. Scale bar = 10 μm. **(C)** Schematic representation of a *Tetrahymena* cell. The longitudinal ciliary rows, containing the aligned basal bodies (BBs), are organized in a polarized and asymmetrical pattern defining a permanent antero-posterior axis and a left-right asymmetry. Associated with each basal body (BB) are the transversal microtubules (TM) and post-ciliary microtubules (PM), as well as the longitudinal microtubules (LM) at their right. The oral apparatus (OA), cilia, contractile vacuole pores (CVPs), cytoproct (Cyp) and the apical crown (AC) are also visible or indicated. When cells initiate division a new oral apparatus (nOA) primordium starts to assemble. Conventional numbering of ciliary rows is indicated in the scheme; rows with the lowest number (1) and highest number (n) are attributed to the two post-oral BB rows. The circumferential asymmetry of the cell is specified. Scheme adapted from Wloga and Frankel (2012). **(D)** Schematic representation of a *Paramecium* cell. As in *Tetrahymena* the longitudinal ciliary rows, containing the aligned basal bodies (BBs) and cilia, are organized in a polarized and asymmetrical pattern defining a permanent antero-posterior axis The oral apparatus (OA), composed by the gullet (G) and oral groove cilia (OG), is present as well as the two contractile vacuoles (CVs). Cytoproct (CYT) and the trychocysts (T) are also visible or indicated.

**FIGURE 2:**
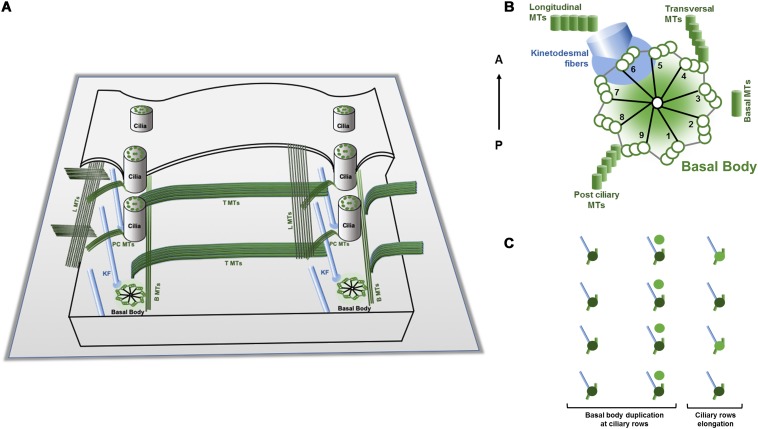
Schematic representations of a *Tetrahymena* cortex, basic cortical unit structures and duplication mode. **(A)** *Tetrahymena* cortex presents a specific pattern of ciliary units oriented in an antero-posterior arrangement. Each unit contains a basal body that assembles a cilium and nucleates two structures of microtubules, the post ciliary (PC) and the transverse (T) microtubules (MTs) ribbons, and the anterior non-microtubule striated fiber designated by ciliary rootlet or kinetodesmal fiber (KF) (see text). Bands of longitudinal microtubules (LMTs) and basal microtubules (BMTs) are running in parallel to both sides of the basal bodies. **(B)** The basal bodies post ciliary microtubules, the transverse microtubules and the rootlet are assembled in association with specific triplets of the centrioles. The rootlet runs anteriorly and at the right side of the basal body and of the cell, whereas the post-ciliary and the transverse microtubule bands run to the left and posterior side of the basal body. These associated basal bodies structures create additional local intrinsic polarities/asymmetries since they present a specific orientation relatively to the antero-posterior axis of the cell (Based on Wloga and Frankel, 2012). **(C)** During division, the cell grows throughout the antero-posterior axis essentially by the addition of new basal bodies and associated structures to the preexisting longitudinal rows. The new assembled basal body maintains the mother arrangement; it is assembled at its anterior right side and then is inserted into the ciliary row and starts to maturate acquiring the appendages as ciliary row elongates (adapted from Frankel et al., 1981; Beisson, 2008).

*Tetrahymena* and *Paramecium* BBs are both structurally and molecularly conserved with the BBs of other Eukaryotes. They are composed of typical triplet MT blades arranged in a radial symmetry giving the BBs its standard barrel shape. However, these BBs present at their proximal region the cartwheel structure that is retained throughout the BBs life. This structure in vertebrates is lost in centrioles/BBs upon their maturation (Azimzadeh and Bornens, 2007; Strnad and Gönczy, 2008). In addition, *Tetrahymena* BBs present two layers of dense material, the terminal plate, that cap the BBs at the distal region (for review see Bayless et al., 2016). Interestingly, in *Paramecium*, not all BBs exhibit the same length; they vary from 330 to 600 nm, with the largest ones being present at the OA. Notably, the size of the cartwheel follows the length of the BBs as a whole. The transition zone in these BBs is organized in three distinct plates, the terminal, the intermediate and the axosomal plate (for review see Tassin et al., 2016). In both ciliates the most distinctive features of the BBs are their accessory structures. These structures comprise the post ciliary and the transverse MTs band, and an anterior striated fiber cytoskeleton-like structure designated by ciliary rootlet or kinetodesmal fiber (see Figures 1, 2) (Allen, 1967; Frankel, 2000; Yang et al., 2002; Lynn, 2008). They help to position, anchor, and coordinate the BBs at the cortex of these ciliates (Bayless et al., 2016; Tassin et al., 2016).

**FIGURE 3:**
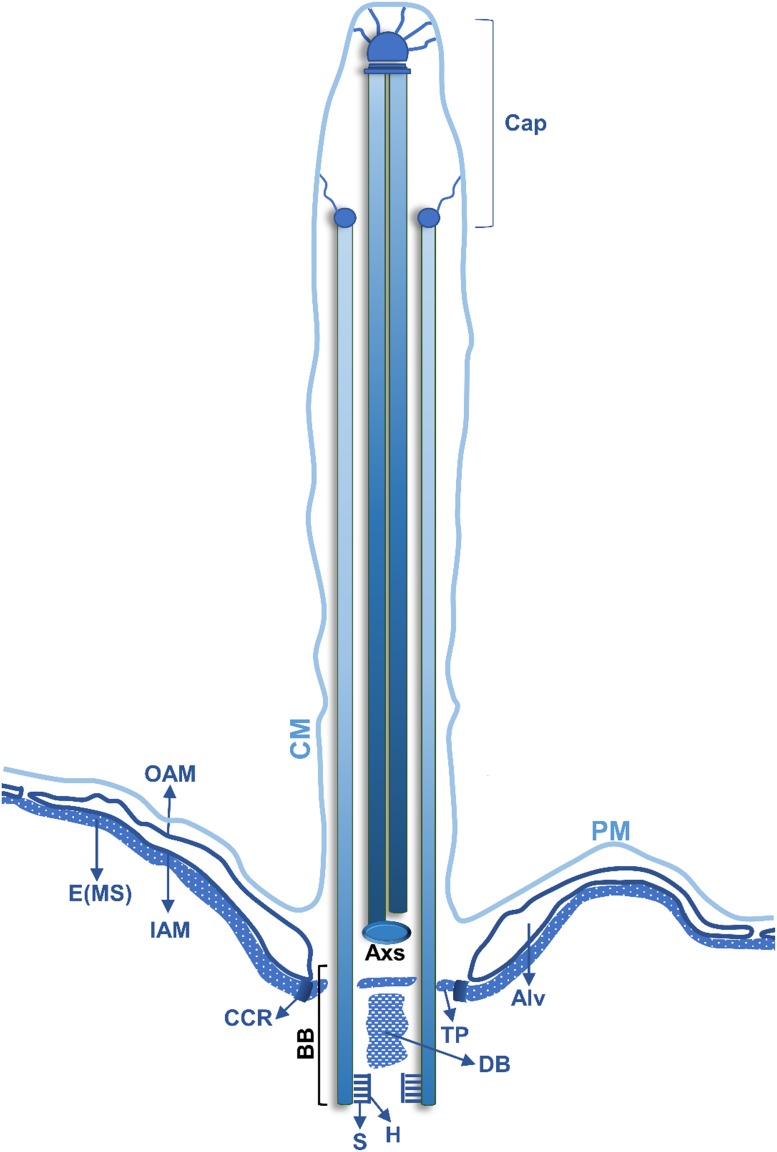
Schematic representation of a longitudinal section of a *Tetrahymena* somatic cilia. The juxtaposed layers constituted by the plasma membrane (PM), in continuity with the ciliary membrane (CM), alveolus (Alv) and the epiplasm (E) are indicated (see text for details). The outer alveolar membrane (OAM) and the inner alveolar membrane (IAM) surround the Alv. In the BB different structures are indicated: the hub (H) and spokes (S) of the cartwheel structure, a dense body (DB) and the terminal plate (TP). A circumciliary ring (CCR) between the TP and the MS. The peripheral MTs (P) of the axoneme are in continuity with 18 of the 27 MTs of the BB. One MT of the central pair MTs (C) of the cilium originates at the axosome (Axs) at the base of the cilium, while the other originates slightly above the Axs. Toward the distal end, the *Tetrahymena* cilia axoneme loses the (9 + 2) pattern, and the peripheral MT doublets become singlets. The singlets maintain a circular positioning and are attached to the cilia membrane through filaments that terminate in plug like structures that are inserted in their lumen. The central MT pair is present until the end of the cilia tip and terminates in a complex cap structure (central MT cap), which also links them to the cilia membrane (adapted from Frankel, 2000).

Despite the size and number differences within both ciliates, most of the BBs assemble motile cilia that are involved in locomotion (the somatic cilia), in food capture (cilia of OA) and signaling transduction (Jennings, 1899; Bloodgood, 2010). Somatic cilia axonemes present a canonical architecture of 9 stable MT doublets radially arranged around a central pair of singlet MTs (9 + 2) (Allen, 1968). The MT doublets are composed of a complete (13 protofilaments) A-tubule and an incomplete B-tubule (10 protofilaments) (Nicastro et al., 2006). Cilia motility is associated with the presence of axoneme-associated structures, such as outer and inner dynein arms, the nexin-dynein complex, which regulates the activity of the dynein arms, and the radial spokes (Satir and Christensen, 2007; Goetz and Anderson, 2010). Toward the distal end, the *Tetrahymena* cilia axoneme loses the (9 + 2) pattern, and the peripheral MT doublets become singlets (A-tubules) through the loss of the B-tubule. The singlets preserve their circular orientation and are attached to the cilia membrane through filaments that terminate in plug like structures that are inserted in their lumen (Figure 3). The central MT pair is observable until the end of the cilia tip and terminates in a complex cap structure (central MT cap), which also links them to the cilia membrane (Figure 3) (Dentler, 1980, 1984).

In *Tetrahymena*, MT functional diversity is generated by the expression of one α-tubulin and two β-tubulin genes (Barahona et al., 1988; Gaertig et al., 1993) and three α-like and six β-like tubulins genes (Eisen et al., 2006; Coyne et al., 2008). These genes are used to assemble subsets of MT structures with different cellular functions. For example, in *T. thermophila* GFP-BTU2 is found in somatic cilia and BBs, whereas GFP-BLT1 and GFP-BLT4 are observed in macronucleus MT arrays and in the mitotic spindle of the micronucleus (Pucciarelli et al., 2012). In *Paramecium* the tubulin gene family possesses two α- and three β-tubulin genes (Dupuis, 1992; Dupuis-Williams et al., 1996; Ruiz et al., 2004).

Besides α- and β-tubulin, other conserved tubulins are encoded by the *Tetrahymena* and *Paramecium* genomes, such as γ, δ, and ε tubulins. In general these tubulins are critical for BBs and cilia structure and function. γ-tubulin is required for duplication and maintenance of BB structure (Ruiz et al., 1999; Shang et al., 2002, 2005). In the case of ε-tubulin this protein is essential for cell survival and its depletion causes the loss of B- and C-tubule of the BBs triplets (Dupuis-Williams et al., 2002). *Paramecium* δ-tubulin depletion causes the loss of the C-tubule of the BBs without affecting the ability of these structures to assemble cilia, but causing the loss of BBs accessory structures (Garreau de Loubresse et al., 2001). This suggests that the C-tubule is required for the binding of the associated structures to the BB. The most divergent η-tubulin plays a role in BB duplication, and its depletion causes the delocalization of γ-tubulin (Ruiz et al., 2000).

Additionally, in *Tetrahymena* and *Paramecium*, tubulin biochemical diversity is amplified by tubulin post-translational modifications like acetylation on α-tubulin, and the polymodifications, glycylation and glutamylation, on α- and β-tubulin (for extensive review Verhey and Gaertig, 2007; Wloga and Gaertig, 2010). In *Tetrahymena*, α-tubulin can probably undergo cycles of tyrosination/detyrosination (Redeker et al., 2005). In this ciliate, the patterns of glycylation and glutamylation are similar since most of the MTs are simultaneous glutamylated and glycylated. In the case of glutamylation, the maximum number of glutamyl units is specific of each distinct MT. Importantly, the two polymodifications are not uniformly distributed neither on MT structures nor on single MTs. For example, cilia axoneme MTs, particularly those of the radially arranged doublets are glutamylated, glycylated, and show lysine acetylation (Gaertig et al., 1995; Xia et al., 2000; Wloga et al., 2008, 2009; Suryavanshi et al., 2010). Axoneme assembly seems to require glutamylation and glycylation since excess or deficiency in these post-translational modifications cause abnormal cilia length (for review Wloga and Frankel, 2012).

In this review, we intend to give an overview of the relationship between cilia, BBs and associated structures, and other prominent MTs based complex structures/organelles and the establishment of the cortical pattern and local and global polarities of the ciliate cell. The mechanisms and signaling pathways involved in the perpetuation of this complex pattern and axiation of ciliate cell will also be under focus.

### The Cortex of Ciliates: Major Cortical Structures and Organization

Ciliates present an elaborated organization of different cytoskeleton arrays at the cortical region that show distinct arrangements in different species. However, in all of them specific patterns of ciliary units (kinetids) can be observed. Each of these units is characterized by a BB (kinetosome) that, in general, assembles the axoneme of motile cilia. In the case of *Tetrahymena* and *Paramecium*, the kinetids are arranged longitudinally in rows that are parallel to the antero-posterior axis of the cell constituting ciliary rows (as in Figures 1, 2). The BBs associated structures present asymmetrical localization relative to the BB structure and a specific orientation relative to the antero-posterior axis of the cell (Figure 2). In fact, the rootlet/kinetodesmal fiber projects anteriorly, whereas the post ciliary MTs band orients posteriorly and the transverse MTs band is perpendicular. Therefore, in each ciliary row, the aligned BBs display the same orientation, and the kinetodesmal fiber of one BB interacts with the post ciliary MTs band of the one anteriorly positioned (Allen, 1969) (Figure 2). In *Tetrahymena*, bands of longitudinal MTs are observed extending at the right side of ciliary rows beneath the cell membrane (Figures 1, 2). In other ciliates, like in *Didinium*, kinetids are restricted to circular regions of the cell, whereas in *Stylonychia* and *Euplotes* they can also group together originating tufts of cilia called cirri (Lynn, 2008).

**FIGURE 4:**
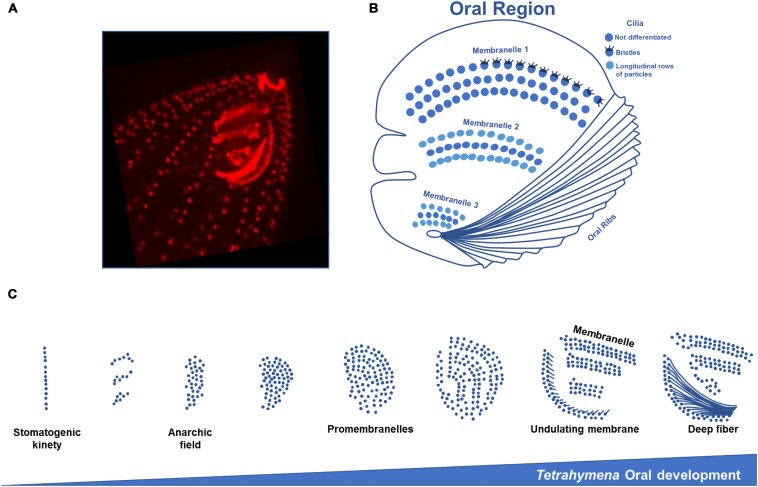
Structure and oral development of *Tetrahymena* oral apparatus. **(A)** Immunofluorescence microscopy using an antibody against centrin of a *T. thermophila* exponentially growing cell showing the three membranelles and ribs of the oral apparatus. **(B)** Scheme of *Tetrahymena* oral region representing the three rows of cilia that compose the membranelles. In the most outer row the first membranelle some cilia present bristles. In the second and third membranelles longitudinal rows of particles are found on the outer rows of cilia. **(C)** The assembly of a new OA initiates with BBs division in an apparent disorganized mode originating an “anarchic field,” next to the right post-oral ciliary row that ends near the posterior margin of the old OA, the “paroral kinety” (row n° 1). Disorganized basal bodies will progressively organize in rows. The acquisition of post-ciliary MTs by the BBs in the anarchic field induces their orientation and alignment, originating four rows. This may result from constrains imposed by the interactions between transverse MTs, post-ciliary MTs and MTs of the paroral kinety with the new assembled BBs. The post-ciliary MTs play a critical role in BB orientation and OA morphogenesis (schemes adapted from Sattler and Staehelin, 1974; Frankel, 1991).

The cortical organizational BBs/ciliary units pattern is disrupted in specific regions of ciliate cells by other BBs configurations originating complex structures as the OA. This structure, specialized in food capture and phagocytosis, is in general composed of membranelles organized from rows of BBs positioned in precise patterns dependent on the ciliates species (Lynn, 2008). For example, in *Tetrahymena* the OA localizes near the anterior pole of the cell and is composed of four membranelles where BBs, in a precise pattern, are interlinked by a network of MTs and other filaments (e.g., tetrins) (Nilsson and Williams, 1966; Gavin, 1980; Bakowska et al., 1982; Honts and Williams, 1990; Dress et al., 1992; Frankel, 2000) (see Figure 4). Observed from the anterior pole, the cell’s right post-oral row is conventionally numbered as 1 and the enumeration continues clockwise around the cell (see Figure 1) (Frankel and Nelsen, 1981). In *Paramecium* the OA is localized in a mid-ventral position creating the oral meridian that defines an axis of right–left asymmetry clearly visible by a line of dissimilarity in the global arrangement of longitudinally BBs rows (Allen, 1988; Beisson, 2008).

In the anterior *Tetrahymena* pole, all the ciliary rows, except those two that are post-oral (Figure 1), end in units composed of a pair of BBs (dikenetids) that originates a characteristic structure of the anterior pole of the cell structure that is designated by the “asymetrical apical crown” (McCoy, 1974; Jerka Dziadosz, 1981). In *Tetrahymena* the more anterior BBs of the apical crown dikinetids are not ciliated (McCoy, 1974; Jerka Dziadosz, 1981), as well as the BBs of the inner row of the OA undulating membrane (Nilsson and Williams, 1966; Gavin, 1980; Bakowska et al., 1982; Dress et al., 1992). Transiently non-ciliated BBs may be observed in central and posterior regions of *Tetrahymena* cells (Frankel, 2000 review). Interestingly, in *Paramecium*, ciliary units comprised of two BBs are more predominant than monokinetids (Iftode et al., 1989).

Although the kinetids can be assumed as the basic units of the cortex structural pattern, other structures adopt specific organized localizations at the cell surface, as for example the cytoproct (CYT) and the contractile vacuole pores (CVPs). In *Tetrahymena* the CYT localizes in the posterior pole of the cell along the ciliary row designated as n°1 (see Figure 1), whereas, in general, two CVPs are also posteriorly positioned to the cell′s right of the oral-cytoproct meridian (near ciliary rows number 5 and 6) (Elliott and Bak, 1964; Loefer et al., 1966; Nanney, 1966b, 1972; Ng and Frankel, 1977; Frankel, 2000; Wloga and Frankel, 2012). The CYT plays an important role in the ejection of the contents of old food vacuoles previously formed at the OA. The CVPs are surface open pores of a single contractile vacuole that in *Tetrahymena* is posteriorly positioned, unlike in *Paramecium*, which contains two contractile vacuoles that are localized at the middorsal region along the same row of BBs, each of which open in a CVP (Allen, 1988). The contractile vacuole is an osmoregulatory organelle that can undergo cyclic accumulation (diastole) and expel (systole) of water, which allows cells to survive under hypotonic conditions. The structure of CVPs is closely associated with MTs that are acetylated and extend from the vacuole pore wall to the vacuole membrane. Proteins associated to the cytoskeleton like γ-tubulin, centrin, calmodulin and Nima-related kinases Nrk1p and Nrk2p also seem to localize at the CVPs (Wloga and Frankel, 2012). Interestingly, in *Paramecium*, the silencing of the gene encoding stomatin, a protein associated with the contractile vacuole complex, causes reduced mechanosensitivity (ciliary reversal upon touching an obstacle) (Reuter et al., 2013).

### The Epiplasm

Basal bodies and associated structures are anchored to a fibrous or filamentous layer that constitutes a submembrane skeleton, the epiplasm (Collins et al., 1980; Lynn, 2008; Aubusson-Fleury et al., 2013). This layer presents different architectures in the different ciliate phylogenetic groups and lies beneath the cortical alveoli (a mosaic of flattened membrane-bounded sacs) that are under the plasma membrane. The alveoli seem to be involved in the Ca^2+^ sequestration/release used in cilia beating coordination (Plattner, 2015). In *Paramecium*, the surface architecture is much more complex (Allen, 1988; Frankel, 2000). The juxtaposed layers constituted by the plasma membrane, alveolus and the epiplasm form a pattern of hexagons and parallelograms over the cell surface. In fact, in *Paramecium* the epiplasm is a thin dense layer segmented into regular units where BBs are implanted forming a hexagonal pattern. In *Tetrahymena* the epiplasm is continuous but presents regions with different flexibilities, corresponding to the localization of the somatic BBs, OA, CVPs and CYT, which are probably related to distinct fibrous network organization (Williams et al., 1987, 1990, 1995). *Paramecium* possesses an additional system of microfilaments, non-bound to membranes, that originates a contractile lattice at the level of the BBs, designated as the infraciliary lattice (Allen, 1988). A large sub-family of centrins, small calcium-binding EF-hand proteins, is a component of the infraciliary lattice, together with a small number of other polypeptides. They constitute branched microfilaments bundles organized in polygonal meshes around the BBs (Klotz et al., 1997). Thus, the ciliate cortex can be viewed, as proposed by Fleury et al. (1992) “as a device for supporting surface elements, i.e., basically as an intracellular “shell,” on which the infraciliature is anchored (…).”

Proteins associated with the epiplasm include articulins (Huttenlauch et al., 1998a, b), alveolins (Gould et al., 2008, 2011; El-Haddad et al., 2013), and epiplasmins (Nahon et al., 1993; Coffe et al., 1996; Bouchard et al., 2001; Pomel et al., 2006). In *Paramecium*, 51 genes encoding epiplasmins were annotated. The encoded proteins can be assigned to five phylogenetic groups and three classes that are characterized by distinct structural features and sequential arrangement of their domains (i.e., symmetric groups, an asymmetric or atypic group) (Pomel et al., 2006; Damaj et al., 2009). Epiplasmins present different cellular localization in the epiplasm unit, a low turnover and different timings of deposition during unit duplication (Aubusson-Fleury et al., 2013). Loss-of-function experiments show that epiplasmins play an important role in epiplasm maintenance and organization, which is crucial for cell division and morphogenesis (Damaj et al., 2009; Aubusson-Fleury et al., 2013). Interestingly, the terminal plate of BBs also contains epiplasmins (Tassin et al., 2016). In *Tetrahymena*, the epiplasmin family seems to be comprised by four members that present a relationship with *Paramecium* phylogenetic groups and share the same type of structural organization (Damaj et al., 2009). They also play a role in shape and cortical pattern (Williams, 2004) and participate in the morphogenesis of OA (Sattler and Staehelin, 1979). Therefore, the epiplasm and, in the case of *Paramecium*, the infracilliature originate, together with BBs, associated structures and cortical MTs, an elaborated cytoskeleton at the cell cortex. This allows cells to maintain shape, but also to be deformable in high viscous media or to contract/extend as in the case of *Didinium*.

### Cilia Diversity in a Single Cell: The Case of *Tetrahymena*

Ciliates offer excellent opportunities to address a myriad of questions concerning BBs and cilia biology, not only because their cells contain incredibly high numbers of these complex structures, but because they present diversity inside a single cell.

In *Tetrahymena*, most of the available information suggests that somatic cilia are structurally similar. However, Wloga and Frankel (2012) have noticed that at the anterior region of the cell most cilia are slightly smaller than those in the middle and posterior regions. Moreover, the length of cilia seems to be regulated by the NIMA-related kinases (NRKs) and the response of individual cilia to the activity of specific NRKs is dependent on the subcellular localization. Therefore, in a single cell the length of specific cilia subsets is differentially regulated by distinct NRKs (Wloga et al., 2006).

Remarkably, the starvation of the ciliate *Tetrahymena pyriformis* and *T. thermophila* dramatically affects cell shape, their cortical features, and causes the emergence of a long posterior cilium of about 15–20 μm. In contrast, a somatic cilium has in average about 7–10 μm (Nelsen, 1978; Nelsen and Debault, 1978). These cells become faster swimmers and this alteration is accompanied by an increase in somatic BBs/cilia number and by the positioning of the oral membranelles beneath the cell surface (Nelsen, 1978; Nelsen and Debault, 1978). In these transformed cells, most unciliated BBs normally detected, are not observed anymore suggesting that the phenotypic transformation involves the assembly of new cilia (Nelsen and Debault, 1978). This shows that the unciliated BBs found in ciliate cells are capable to nucleate and assemble an axoneme.

These observations clearly indicate that the nutritional state of the cell, and probably other environmental alterations, can induce, not only the changes in the number of cilia/BBs, but also signaling to unciliated BBs to assemble somatic cilia and a unique long cilium. The ability to respond to environmental challenges with morphological alterations, including cilia/BBs, is not an exclusive feature of *Tetrahymena* species. In *Paramecium*, the somatic and oral cilia differentially respond to changes in their mechanical environment characterized by modifications in media viscosity (Jung et al., 2014). As far as media viscosity increases the somatic cilia beat frequency decreases nearly inversely proportional to viscosity, which allows somatic cilia to generate a constant propulsion force. In contrast, viscosity increasing does not affect the OA cilia beat frequency. This strongly indicates that both types of cilia should have different mechanisms controlling cilia beating (Jung et al., 2014) and/or different capabilities to sense viscosity and adjust motility. Moreover, in the ciliate *Dileptus anser* the sensory cilium undergoes an additional formation of some structural elements and dedifferentiation of the others, being transformed into a motile cilium. This resembles the capacity of certain *Tetrahymena* kinetosomal couplets of oral origin to form additional rows of motile cilia in nutrients’ privation (Nelsen and Frankel, 1979).

The ability of cilia to adapt/respond to environmental alterations by modifying their sensory capacities, or even their architecture, is not exclusive of ciliates but is still poorly understood. For example, in *Caenorhabditis elegans*, cilia are able to alter their length, specifically that of the most distal domain in response to external stimuli, as for example the lack of sensory signaling and hypo- or hyper-osmotic alterations (Mukhopadhyay et al., 2008; Rich and Clark, 2012). During the mating process of the algae *Chlamydomonas* the flagella tips change their structures increasing the tip segment length by 30% and accumulating adhesive complexes (agglutinins) which seem to be required for conjugation signaling (Mesland et al., 1980; Goodenough, 1993).

The analysis of the ciliate cell organization above reported clearly shows that, in *Tetrahymena* and *Paramecium*, the BBs and associated structures, longitudinally organized in rows through the antero-posterior axis of the cell, are the core of cortex basic structural repeating units. The fact that BBs associated structures (Figure 2) present a specific orientation relative to the antero-posterior axis of the cell and an asymmetric positioning to the BB structure locally creates intrinsic polarities/asymmetries. Therefore, each structural unit contributes to the global and local organization of the cortex. This organization not only shapes the cell but impacts on how other structures and organelles are distributed. This cortex structural pattern is broken in specific regions by the occurrence of new organization patterns of BBs as those of the apical crown and the OA, or by the complex structures associated with the MT cytoskeleton of the CYT and CVPs. Consequently, in both ciliates the cortical asymmetrically located structures associated with the asymmetry at the level of each BB originates two orthogonal directions of cell polarity, an antero-posterior (corresponding to the swimming direction of the cell) and a circumferential (Frankel, 2000; Beisson, 2008).

Another degree of complexity in ciliate cortex is the possibility that ciliary units are not completely structurally/functionally identical and different populations may coexist in the cell. This idea is supported by the existence of *Tetrahymena* cilia structural/functional diversity and simultaneously by the observation that some cilia/BB show the ability to respond to variations in their environment. This response is complex and not global since it can be regulated throughout different regions/structures of the cell/organism showing the existence of specific territories.

How such an elaborated pattern of organization is perpetuated in an organism that divides symmetrically producing two tandemly arranged daughter cells of identical polarity creates a development problem. This requires not only the duplication of the complex cortical structures, but also their correct positioning and eventually their differentiation. This complexity and how it is perpetuated has lead Beisson (Beisson, 2008) to state that ciliate cortical organization is “equivalent of multiple different organs, arranged in a specific body plan so that each division involves complex morphogenetic movements akin to developmental processes.” Thus the pertinent question is: what are the molecular mechanisms that allow a single cell-organism to rigorously control in space and time the maintenance of this complex structure when cells divide? We intend to review the current knowledge that contributes to answer this question in the following sections.

## Perpetuation of a Complex Pattern Within a Single Cell

The complex ciliate cortical organization pattern is perpetuated during cell division, which, in *Paramecium* and *Tetrahymena*, occurs by binary fission. During this process a fission furrow develops at the equatorial region of the cell causing a break in the continuity of longitudinal rows of cortical units. Essentially, the addition of new BBs to the preexisting longitudinal rows causes the growth of the cell throughout the antero-posterior axis (Iftode et al., 1989; Frankel, 2000; Wloga and Frankel, 2012). In growing *Paramecium* the new BB is assembled at the anterior right side of the old BB and then is inserted into the ciliary row, remaining aligned with the old BB (Figure 2) (Beisson and Jerka-Dziadosz, 1999). In *Tetrahymena* the process is similar, and the newest BB usually does not possess transverse MT bands that only start to assemble when it separates and reaches a certain distance from the old BB (Nelsen et al., 1981). The correct distance between the new BB and the old BB seems to be dependent on the kinetodesmal fiber of the old BB (Allen, 1969). The position of each new BB is therefore conditioned by the old pre-existing BB (Beisson and Jerka-Dziadosz, 1999), namely by the asymmetric localization of the old BB accessory structures.

**FIGURE 5:**
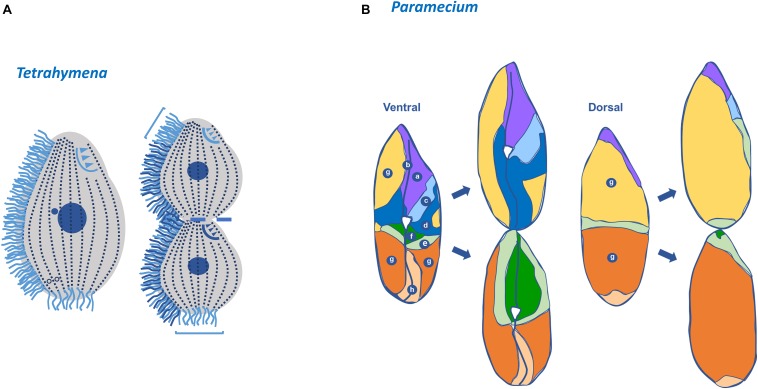
*Tetrahymena* invariable regions and *Paramecium* fate territories. **(A)** In *Tetrahymena*, the most posterior region of the posterior daughter cell (opisthe) and in the most anterior region of the anterior daughter cell (proter) the basal bodies and cilia are those that are maintained for several generations. In the dividing cell: (i) the new formed basal bodies, cilia and oral apparatus (dark blue); (ii) the old basal bodies, cilia and oral apparatus (light blue) (adapted from Thazhath et al., 2004). Invariant regions are also present in *Paramecium*. **(B)** The distinct territories in the mother cell are defined based on the timing and the degree of basal body proliferation during division (a,b,h) correspond to invariant fields that are inherited linearly; (g) in daughter this field will expand longitudinally and laterally; (f) expansion of a small ventral field that will be invariant in the next division (d,e) these two fields undergo a process of expansion and repositioning to the posterior and anterior end, respectively; (c) this field suffers a huge expansion toward the anterior pole (*Paramecium* scheme adapted from Iftode et al., 1989).

In *Tetrahymena*, transitory unciliated BBs can be found more frequently in the middle and posterior region of the cell (Nanney, 1975; Nelsen et al., 1981). In fact, cilia only emerge on BBs where transverse MT bands already achieved their full length (Frankel et al., 1981). These observations suggest the existence of a delay between the initial assembly of new BBs and their ability to assemble an axoneme. This period of maturation seems to be one cell cycle (Nanney, 1975; Frankel et al., 1981). In these regions, a more irregular spacing between BBs is also observed (Frankel and Nelsen, 1981). Interestingly, the appearance of new structurally different BBs, with distinct functional competences also increases the local polarity complexity (Beisson and Jerka-Dziadosz, 1999). Moreover, after cytokinesis each daughter cell will possess BBs with different ages and maturation states. In *Tetrahymena*, the BBs and cilia in the most anterior region of the anterior daughter cell and in the most posterior region of the posterior daughter cell are those that are maintained for several generations, which may impinge specific features to these conserved regions. These regions are also present in *Paramecium* and are usually designated as invariant regions (Figure 5) (Iftode et al., 1989; Thazhath et al., 2004).

In *Paramecium*, the cortical units are similarly organized and roughly of the same size but they differ in respect to the number of BBs that they contain and to their contribution for the formation of daughter cells. Therefore, in each cell it is possible to define different fields that are characterized by units containing a unique BB, two BBs or fields where both types of the units co-exist. The duplication of these different types of BBs fields is complex and occurs in two waves of proliferation (see Iftode et al., 1989 for details). Moreover, intercalated with these fields there are regions where no BB duplication occurs. The most anterior of these regions will be inherited by the anterior sibling, whereas the most posterior by the posterior daughter cell (Iftode et al., 1989). Therefore, the *Paramecium* cortex is a mosaic of territories that differentially contribute to the cortex of the two daughter cells (Figure 5) (Jerka-Dziadosz and Beisson, 1990). However, the different contribution of each field to the progeny cortex seems to reside in their localization and not in the distinct properties of particular units (Iftode et al., 1989).

In *Tetrahymena* and *Paramecium* the assembly of a new OA initiates with BBs proliferating in an apparent disorganized mode, originating an “anarchic field.” This event starts next to the right post-oral ciliary row that ends near the posterior margin of the old OA (the “paroral kinety”; row numbered as n° 1 in Figure 1) (Chatton et al., 1931; Frankel and Williams, 1973), but present differences between the two species. Contrary to *Tetrahymena*, in *Paramecium* the new OA originates from the parental anarchic field that was assembled in the last division. Consequently, the parental post-oral ciliary row does not participate in the formation of the oral primordium but renews the anarchic field that will be used in the next cell division (for details see Iftode et al., 1997). In both species the “anarchic” organization of the BBs is a transitory stage since they will progressively organize in rows. Although the somatic BBs arise by a “template” pathway based on the old BBs, the biogenesis of OA BBs may rely in a “*de novo*” pathway (for review Bayless et al., 2016). The BBs of the developing OA of *Tetrahymena* and *Paramecium* contain MT post-ciliary fibers, but not kinetodesmal fibers (Frankel and Williams, 1973). In *Tetrahymena*, it was proposed that the BBs progressively organize in space under the action of two vectors (Frankel, 1991). One is oriented from posterior-left to anterior-right of the cell, and was designated by Frankel (1991) as the “global field vector.” The second vector, designated by “geometry vector,” points into the same direction in different BBs and reflects their intrinsic polarity (i.e., posterior-right represents the post-ciliary MT band). In *Paramecium*, the mode of BB duplication during the first wave in the cortex and in the oral field is similar, and originates a pre-patterning (Iftode et al., 1997). The acquisition of post-ciliary MTs by the BBs in the anarchic field induces their orientation and alignment, originating four rows. Therefore, in both ciliates, post-ciliary MTs play a critical role in BB orientation and OA morphogenesis. This may result from constrains imposed by the interactions between transverse MTs, post-ciliary MTs and MTs of the post-oral ciliary row with the new assembled BBs (Iftode et al., 1997), as well as underlying MTs networks. Interestingly, in *Tetrahymena* and *Paramecium* the development of the OA is coordinated with the BB assembly and ciliation in somatic ciliary rows suggesting the existence of a morphogenetic coordinating mechanism that integrates both events (Nelsen et al., 1981; Iftode et al., 1997). In both ciliates the OA BBs alignment occurs through the guidance of the antero-posterior and circumferential cell axes in close cross-talk with the local asymmetric structures of BBs and associated structures (Frankel, 1991).

The importance of the positional information in cortical patterning perpetuation is also evidenced by the way new CVPs are positioning in siblings (Nanney, 1966a). In *Tetrahymena* the new CVPs are assembled anteriorly to the old ones in a cortical domain defined longitudinally by the oral meridian (see Figure 1), and the CVP meridian that extends from the anterior pole of the cell to the midpoint of the old CVPs. A correlation between the number and the position of CVPs and the total number of ciliary rows seems to exist (Nanney, 1966b, 1972). Therefore, these structures are positioned at the right of the oral meridian at a distance proportional to the cell circumference (Figure 1) (Frankel et al., 1993). The information residing in this domain allows the formation of new structures along the same longitudes as the corresponding old ones (Frankel, 1992).

Concluding, the symmetric division of *Paramecium* and *Tetrahymena* reconstitutes the mother cortex patterning in each sibling. This reconstitution seems to involve a continuous cross-talk between two types of mechanisms: those operating locally and those controlling the cell global axes of polarity (Jerka-Dziadosz and Beisson, 1990). Local mechanisms would involve BBs and accessory structures and are probably based on a pre-pattern relying in the existence of a guiding scaffold or nucleation points. The ciliates cytoskeleton and the epiplasm emerge as potential critical players in this mechanism. The global mechanisms involving these structures would control the establishing of the cell global axes of polarity during cell division and would depend on the propagation of inductive signals that are differently read by the distinct morphogenetic territories, e.g., the OA cortical domain. Although the positional information and biochemical differences between the distinct territories that confer their identity are far from being completely understood, some progress to understand these mechanisms has been done.

## The Cortical Inheritance Mechanisms

### The Cytotaxis Concept

Pioneering work of how the ciliate cell patterning is maintained during cell division was initiated with the study of *Paramecium* doublets. This type of cells appears spontaneously in cultures and they are stably perpetuated throughout several generations. Doublets are characterized by possessing two complete sets of organelles and BB fields arranged in tandem and seem to arise during abnormal ending of conjugation (Beisson, 2008). In *Paramecium* conjugant pairs, each cell stays with a gametic nucleus and its cytoplasmic information, and receives a gametic nucleus from the other conjugant partner. After these nuclei exchange, the two gametic nuclei fuse in each conjugating cell originating two F1 progeny clones. Both clones possess identical heterozygous nuclear genotypes but maintain their specific cytoplasmic information. During this event, conjugant cells establish cytoplasmic bridges that are abscised at the end of the conjugation (Beisson, 2008). Sometimes abscission fails and the two conjugants remain fused, originating a doublet cell. When doublets are crossed with normal partners the phenotype is maternally inherited and clonally stable, suggesting the existence of a “cortical inheritance” (for review Faureé-Fremiet, 1948; Jerka-Dziadosz and Beisson, 1990; Beisson, 2008). Moreover, siamese-twin doublets microsurgically constructed in the large ciliate *Stentor coeruleus* are also able to propagate their doublet morphology (Tartar, 1956). This idea was consolidated by Beisson and Sonneborn (1965), which conducted a very elegant experiment where a piece of cortex of one *Paramecium* cell, after a rotation of 180°, was grafted on another *Paramecium* cell. The authors observed that, in the grafted cell, the inverted cortical BB rows were perpetuated in the following generations, although the new cells were genotypically identical to their parents. The cilia assembled by the inverted BBs beat in opposite direction to that of normally oriented cilia (Tamm, 1975). Clearly, the new cortical pattern organization of the grafted cell was able to determine *per se* the organization of the cortical pattern of the sibling’s cells. This new organization was independent of the overall antero-posterior polarity of the cell. These observations lead Sonneborn (Sonneborn, 1964) to propose the concept of cytotaxis, that he defined as “ordering and arranging of new cell structure under the influence of preexisting cell structure” and proposed to be an important principle in cell differentiation (Sonneborn, 1964). In light of this concept it is assumed the existence of a level of information that resides in the cell structural pattern, controlling its transmission to the daughter cells during development without direct intervention of the genome (Sonneborn, 1964; Beisson and Sonneborn, 1965). Later, Ng and Frankel (1977) confirmed that cytotaxis was not a particularity of *Paramecium* cells since *Tetrahymena’s* 180° rotated ciliary rows were also perpetuated. These rotated rows contain information for the assembly, positioning, orientation of new BBs and associated MT rootlets and kinetodesmal fibers. Thus, the new BB orientation seems to impose constraints to the positioning and arrangement of other structures beyond the local cortical units, impacting in the overall organization of the cell (Ng and Frankel, 1977). For example, the alteration of kinetodesmal fibers and microtubular bands’ orientation impacted in cortex MT cytoskeleton and consequently in the positioning and development of other structures like the CVPs (Ng and Frankel, 1977).

### The Cortical Determinant Regions

The study of ciliates encystment brought new information for the understanding of how the complex patterning of ciliates is perpetuated. The *Oxytricha fallax* encystment is characterized by the disassembly of cortical cilia, BBs and MTs that reassemble within 2 h after excystement, recovering their exact pre-cyst ciliature (Grimes, 1973; Hammersmith, 1976). This is true for normal cells or cells with altered phenotypes, as for example, doublets, cells with inverted ciliary rows or supernumerary dorsal bristle rows (Grimes, 1973; Hammersmith, 1976; Hammersmith and Grimes, 1981). In these different cases, the cell pattern is completely recovered in the absence of visible preexisting structures. Consequently, these studies support the existence of determinant cortical regions that seem responsible for the primordium of ciliature even in the absence of cortical structures (Grimes, 1973). The presence of these determinant regions was previously noticed in studies involving the regeneration of the ciliate *Urostyla grandis* and during the OA development of *Paramecium* (Hanson, 1962; Jerka-Dziadosz, 1964). However, the use of the encystment/excystement system unequivocally showed their presence. More recently, a proteomic analysis revealed that *Euplotes* encystment involve proteins with a wide range of molecular functions, including gene regulation, RNA regulation, proteins degradation and oxidation, resistance, stress response, material transport and cytoskeleton organization (Chen et al., 2014). Still, it remains poorly understood how determinant regions assist patterning recovery.

### The Cortical Patterning Mutants

Another important step in the clarification of the mechanisms that govern ciliates’ patterning was the analysis and characterization of *Tetrahymena* and *Paramecium* mutants occurring spontaneously or generated by the exposure to mutagenic agents. Most of the observed mutants phenotypes could be organized in four major classes: (I) those showing altered organization of BBs in the ciliary rows; (II) those presenting abnormal development and structure of the OA; (III) those with compromised general cell polarity (i.e., with alterations along the antero-posterior and the circumferential axes); and (IV) those showing combinations of these different anomalies (for extended reviews see Jerka-Dziadosz and Beisson, 1990; Frankel, 2008). An illustrative example of the first class is the *Tetrahymena* disorganized-A (*disA-1*) mutant characterized by a single-locus recessive mutation where positioning and orientation of BBs and kinetodesmal fibers are dramatically disorganized (Frankel, 1979, 2008; Jerka-Dziadosz et al., 1995). Still, these cells remain able to assemble new OA and CVPs in a normal localization relatively to the antero-posterior axis of the cells. This indicates that the positioning of OA and CVP does not rely on ciliary rows’ organization and additional information should contribute for their correct localization. Moreover, this mutation does not affect global cellular polarity or ciliogenesis showing that these are dissociated from specific cortical units. Unlike the *disA-1* mutation, the *Paramecium* monogenic nuclear recessive mutation *Kin241* (Jerka-Dziadosz et al., 1992) originates ciliary row inversions and simultaneously affects the structure, the duplication of BBs and the body plan of the cell (Jerka-Dziadosz et al., 1992). Interestingly, the nucleation of a second kinetodesmal fiber was associated to BB mispositioning and misorientation (Jerka-Dziadosz et al., 1992). The boundaries of some cortical domains were also affected which seems to be related to excess of BBs proliferation. According to Frankel (2008) the analysis of several mutations shows that, in certain cases, they may weaken the transmission of preexisting structural phenotypes. This is demonstrated in the case of the monogenic basal-body deficient (bbd) condition in *Euplotes minuta* (Frankel, 1973) and the low kinety number (*lkn1-1*) mutant in *Tetrahymena* (Frankel, 2008). In cells homozygous for the *lkn1-1* mutation, the number of ciliary rows decreased, and their conservation was affected during vegetative growth. Also, the mutants possess incomplete ciliary rows, which are rare in wild-type cells, and their number is propagated with low accuracy. These observations support the idea that the *lkn1-1* mutation dramatically affects the strict cytotactic control of the propagation of ciliary rows (Frankel, 2008).

The mutations that affect OA development, like those that occur in *Tetrahymena* non-phagocytosis (*NP1*) (Williams and Honts, 1987) or *Tetrahymena mpC-1* and *mpC-2* (Frankel, 2008), made evident that there is an independent morphogenetic domain for the OA, and that the patterning of the membranelles is spatially coordinated. Another group of mutations, like the *janus-type* in *Tetrahymena*, evidenced the existence of specific cortical domains that can be individually regulated by cellular signals, and the distinct genetic regulation of the antero-posterior and dorso-ventral polarities. *janus* phenotypes are generated by mutations in three distinct *loci* (on chromosome 3R -JANA (two mutant alleles); chromosome 2 -JANB (one mutant allele) and probably in chromosome 1R -JANC (four mutant alleles) (for review Frankel, 2008). The major phenotype shared by these different alleles is the replacement of the cortical pattern of the dorsal region into a reversed ventral pattern originating two ventral regions that are a mirror-image of each other. Therefore, although mutants present a normal number of ciliary rows an additional OA, partially inverted, is present in the reversed region and two CVPs are found to its left. The extensive analysis of these mutants shows that they are not doublets, but instead singlet cells in which a specific domain suffered a reversal of circumferential polarity (Frankel and Nelsen, 1981; Frankel et al., 1984; Cole et al., 1988). Moreover, in *T. thermophila* a single-gene recessive mutation is able to broaden cortical domains (*bcd*) within which the OAs and the CVPs are assembled and occurs in two locus of the BCD1 gene, *bcd1-1* and *bcd1-2*, located on chromosome 3 (Cole et al., 1988). Similarly, the multi-left-marginal (*mlm*) mutant controlled by a recessive gene of the ciliate *Paraurostyla weissei* (Jerka-Dziadosz, 1989) also causes variable broadening of cortical domains.

Unfortunately, although suggestions and attempts to establish functional relationships between the characteristics of a few known ciliate genes and their mutant phenotypes, in most of the cases, there is still missing a clear correspondence between the mutants and the identity of the genes that are affected. Exception to this scenario is the case of *disA-1*, *cdaI-1*, and *elo1-1* mutations, three mutations that cause unequal cell division due to division plane displacements and deficiencies in nuclear divisions and cytokinesis (Frankel, 2008) that were already assigned to specific genes by using comparative whole genome sequencing (Galati et al., 2014; Jiang et al., 2017). These mutations will be discussed in the next section.

### The Genes and Signals Behind the Ciliates Patterning

#### The Molecules Behind the Asymmetric Nature of Basal Bodies

As discussed, the asymmetric nature of BBs is mostly linked to their associated structures. However, each of these structures is assembled in close association with specific BB centriole triplets, which clearly suggests that distinct proteins should present a differential localization at the BB. An example of this is the protein DisAp encoded by the identified gene responsible for the *disA-1* mutant phenotype (disorganized positioning and orientation of BBs and kinetodesmal fibers) (Galati et al., 2014). DisAp accumulates near the proximal region of the kinetodesmal fibers and is responsible for the kinetodesmal fiber elongation and BB orientation in response to ciliary forces (Galati et al., 2014). This study reinforces the idea that the kinetodesmal fiber is critical for the new BBs positioning at the cortex (Galati et al., 2014).

Another example of proteins involved in BB asymmetry is the centrin family. In *Paramecium* knockdown of the centrin genes CEN2a/2b and CEN3a/3b, orthologs of the *Chlamydomonas* VFL2/human CEN2, and the yeast CDC31/human CEN3, respectively, showed that these centrins are required to define the precise site of the assemble of the new BB and the associated structures (Ruiz et al., 2005). Interestingly, CEN3 is recruited to the connection between the kinetodesmal fiber and the BB by VFL3-A. There, CEN3 is required for the assembly of a transient accessory structure of the old BB, the anterior left filament. During BB duplication this structure guides the migration of the new BB to the cell surface (Jerka-Dziadosz et al., 2013). In *Paramecium*, VFL3-A knockdown affects the correct positioning of the BB by causing the absence or disorientation (pointing in all directions relative to the cellular antero-posterior axis) of the kinetodesmal fibers. In BBs lacking kinetodesmal fibers, the microtubular accessory structures are also absent. Therefore, VFL3-A loss causes alterations in BB intrinsic polarity being involved in the establishment of their rotational asymmetry (Bengueddach et al., 2017).

The proteins associated with BBs stability also contribute for their intrinsic polarity. The protein Fop1 is enriched at the BB posterior face in triplets 1, 2, 8, and 9 (Figure 2) and functionally interacts with BB stabilizers Bld10/Cep135 and Poc1 proteins (Pearson et al., 2009; Bayless et al., 2012, 2016). This protein stabilizes the BB structure from the forces produced by cilia beating and their polarized localization is independent of cilia assembly, but progressively increases with the continuous beating of cilia (Bayless et al., 2016). The depletion of Fop1 causes BB loss unless cilia are inhibited to beat. It is well established that the BB MTs are polyglutamylated through their longitudinal length, which is assumed to stabilize the BB structure (Bobinnec et al., 1998a, b; Wloga et al., 2010, 2017). Interestingly, an asymmetric distribution of glutamylation with a similar pattern of that of Fop1 is observed in *Tetrahymena* BB. Also, Fop1 depletion causes an increase in BB polyglutamylation, whereas the reverse is observed when Fop1 is overexpressed suggesting a compensation mechanism in BB stabilization (Bayless et al., 2016). How the BB stabilizer factors, and MT post-translational modifications patterns play a role in defining local information and impact in patterning and morphogenesis signaling pathways is far from being completely understood.

#### The Molecules Behind the Global Patterning

There are evidences that filaments of unknown biochemical nature may be involved in ciliates patterning determination. In the ciliate *P. weissei* it was proposed that filaments observed in interphase cells or during excystment would be able to constitute scaffolding tracks, and eventually guides for cellular components to be assembled in certain regions (Fleury et al., 1993). These filaments are decorated by the CTR210 antibody that in *Paramecium* recognizes subpopulations of MTs, as for example, the post-ciliary MTs (Fleury et al., 1993), as well as the centrosomes in human cells (Perret et al., 1995). Another antibody, the XXXIX-12G9, obtained against the *Tetrahymena* cortex recognizes filaments that are visible before the proliferation of new BBs in the oral primordium and in the fission line suggesting that the recognized protein is involved in these events (Jerka-Dziadosz and Czupryn, 1997). Data coming from a few mutants support the view that the epiplasm may contain information for pattern organization (Kaczanowska et al., 1995). In *Tetrahymena NPl* mutant cells, the BBs of the anarchic field are unable to orient and attain a correct positioning during OA assembly. In these mutant cells all BBs and MTs present normal structures but are anomalously organized in space. This suggests that the cortex is probably locally differentiated and that the epiplasm has the ability to impinge organizational information (Williams and Honts, 1987).

Cole (2006) noticed that the cortical domains affected by the *bcd* mutation involve fenestrin which led him to propose that the *Tetrahymena bcd* mutation may directly or indirectly affect fenestrin localization. Fenestrin seems to be a ciliate- or even oligohymenophora-specific coiled–coil protein (Cole et al., 2008) of the epiplasm that surrounds the BBs, the OA and the CVPs. Remarkably, during cell division, this protein accumulates in the anterior region and at the fission zone of the cell, suggesting a role in antero-posterior cell axis establishment (Kaczanowska et al., 2003; Joachimiak et al., 2013). In *T. pyriformis*, fenestrin localization pattern and gene expression varies during cell cycle, and the protein is post-translationaly modified, being phosphorylation one of the putative modifications (Joachimiak et al., 2013). Noticeably, fenestrin co-purifies with the Epc1p protein (formerly known as EpiC), one of the three major epiplasmin proteins [epiplasmic band proteins A (EpiA), and B (EpiB)]. Epc1p is a protein evolutionary related with invertebrate intermediate filaments and metazoan lamin proteins and is regulated by phosphorylation (Vaudaux, 1976; Williams et al., 1979; Kaczanowska et al., 1999; Bouchard et al., 2001; Honts and Williams, 2003).

In *Tetrahymena*, the epiplasm protein alveolin ALV2 localizes between the rows of longitudinal MTs and its knockdown causes the loss of cell polarity and blocks cytokinesis (El-Haddad et al., 2013).

The analysis of centrins, in *Paramecium* gave clues that these proteins may have an active role in the maintenance of the ciliate cortical pattern not only al level of the BB asymmetry but also being components and organizing the infraciliary lattice (Klotz et al., 1997). When *Paramecium* cells divide, this network goes through a controlled disassembly especially in the region where the division furrow will be formed. The organizational pattern of this infraciliary lattice is dependent on BBs since each BB contains a specific nucleation site for these filaments that bundle by the preexisting ones (Beisson et al., 2001). Silencing of the genes encoding centrins causes the total disassembly of the infraciliary lattice (Beisson et al., 2001). These data clearly indicate that the local asymmetry of BB is in close cross-talk with infraciliary lattice and this mutual influence is required for local and global information involved in cortical patterning perpetuation.

#### Signaling Pathways Governing Patterning

The occurrence of phosphorylation/desphosphorylation waves spreading across the ciliate cortex were predicted to occur, and to play a role in the signaling that differentially stimulates the different morphogenetic cortical territories. For example, Frankel suggested that “position information” might be based on modifications, such as protein phosphorylation, that quantitatively vary around the cell circumference and are able to propagate from posterior to anterior poles, and vice-versa, during cell division (Frankel, 1991, 1992). In *Paramecium*, during BBs duplication, kinetodesmal fibers undergo a cycle of disassembly reassembly (Fernandez-Galiano, 1978), which is dependent on the hyperphosphorylation of their constituents (Sperling et al., 1991). Moreover, the specific phosphorylation of γ-tubulin is important for either the assembly or stability of BBs (Joachimiak et al., 2018). It is expected that these waves signal over the cell surface to effector systems creating signaling pathways able to regulate cell morphogenesis. The discovery that some components of the evolutionarily conserved Hippo signaling network should play a critical role on morphogenesis and perpetuation of ciliates patterning, gave the first indications of a molecular signaling that operates during these processes.

In metazoans, Hippo signaling pathway has a central role in regulating cell proliferation and cell fate to control organ growth and regeneration (for review Misra and Irvine, 2018). In *T. thermophila* Tavares et al. (2012) showed that depletion of Mob1 caused the abnormal establishment of the cell division plane and cytokinesis arrest. Mob1 is a member of both the Hippo signaling pathway and the mitotic exit network (MEN) that regulates cytokinesis (Hergovich, 2017; Misra and Irvine, 2018). In the core kinase module of the Hippo signaling, Mob1 acts as a central signal adaptor that can interact directly with MST1/2, LATS1/2 and NDR1/2 kinases (for review Misra and Irvine, 2018). Mob1 binds to the highly conserved NTR domain of NDR/LATS kinases and stimulates their activity required to multiple functions (Hergovich, 2011; Sharif and Hergovich, 2018). The Mob1 phosphorylation state regulates its binding to the NTR of NDR/LATS kinases and is dependent on the MST1/2 kinases activity (Kim et al., 2016; Sharif and Hergovich, 2018).

The work in *Tetrahymena* also showed that Mob1 accumulates in the posterior pole BBs, generating a gradient through the antero-posterior axis. *Tetrahymena* Mob1 is recruited to the BBs localized at the cell midzone zone during cell division, just above the region where the cleavage furrow will be formed. Moreover, in dividing cells, it slightly decorates the new OA at the beginning of its biogenesis. Consequently, Mob1 localizes at the new posterior pole of the anterior sibling. This strongly suggests that *Tetrahymena* Mob1 is required for the establishment and maintenance of the antero-posterior polarity and for the correct positioning of the cell division axis. Later, using the ciliate *S. coeruleus*, Slabodnick et al. (2014) confirmed that Mob1 accumulates preferentially in posterior pole and at the midline of dividing cells, being a critical factor in cell polarity establishment apart from its role in cytokinesis. Interestingly, in *Stentor*, Mob1 depleted cells experienced spontaneous OA regeneration in the absence of cell division, suggesting that Mob1 is required for OA localization. Like *Tetrahymena* and *Paramecium*, *Stentor* possesses a complex patterning and axiation but strikingly this ciliate can regenerate an entire cell-organism from a piece of one cell (Morgan, 1901). Taking profit of this feature, they were able to surgically remove Mob1 protein from the anterior or posterior pole of *Stentor* in cells in a Mob1 knockdown background. This allowed them to conclude that Mob1 plays a role in the establishment of both anterior and posterior polarity in *Stentor* (Slabodnick et al., 2014). Both studies showed that morphogenesis is linked to accurate cell division, which is required to maintain cell ploidy and genomic stability (Tavares et al., 2012; Slabodnick et al., 2014).

**FIGURE 6:**
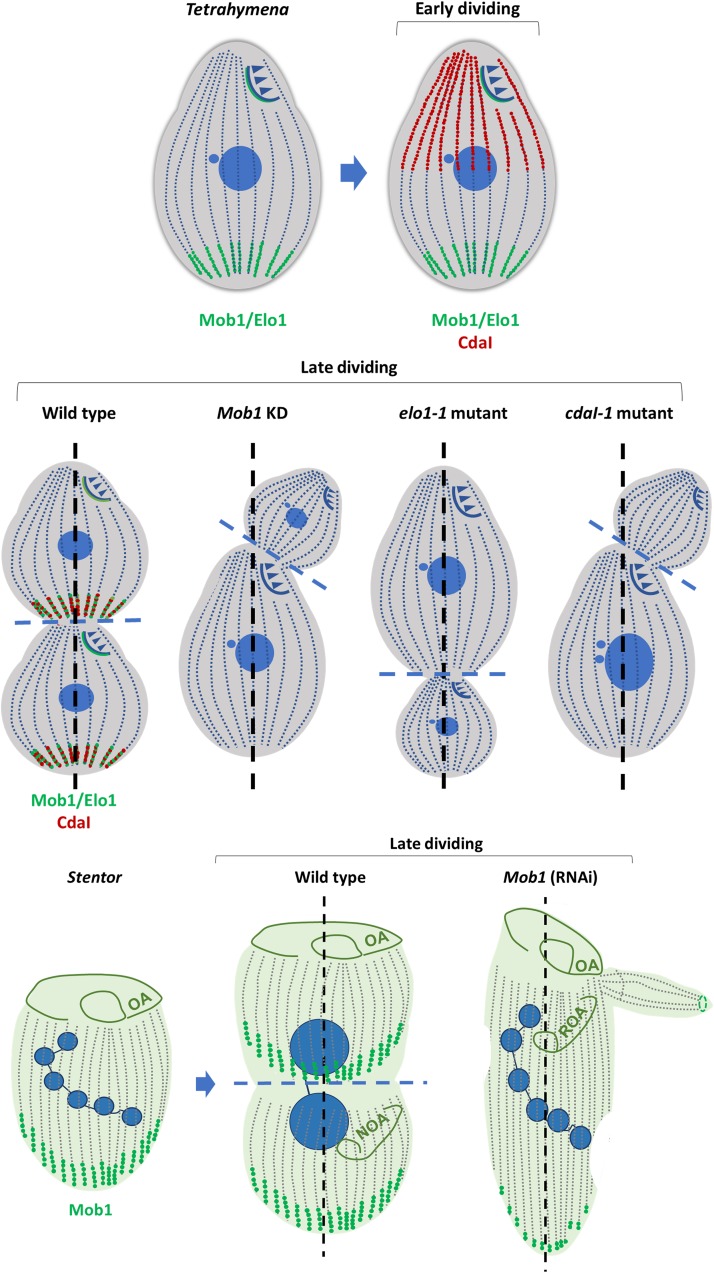
Components of Hippo signaling pathway are players in ciliates cytokinesis and morphogenesis. Mob1 and Elo1 protein localization is shown in green; CdaI protein localization is shown in red. The three proteins show antero-posterior polarized localization. Interplay between Mob1, Elo1 and CdaI regulates the positioning of the division plane and morphogenesis in *Tetrahymena* (Tavares et al., 2012; Jiang et al., 2017, 2019). In *Stentor* Mob1 is known to play a similar role. Depletion of Mob1, Elo1 and CdaI in *Tetrahymena* leads to morphological defects. Depletion of Mob1 in *Stentor* originates similar phenotypes (adapted from Chalker and Frankel, 2014). Anterio-posterior axis is in black dashed line; cell division plane is in blue dashed line. Oral apparatus (OA); new oral apparatus (NOA); regenerating oral apparatus (ROA).

More recently, Jiang et al. (2017) were able to assign the identity of the gene mutated in the *Tetrahymena cdaI-1* mutants (Frankel, 2008), that phenocopies the depletion of Mob1 in *Tetrahymena* (Tavares et al., 2012), to a homolog of the MST1/2 kinases. Interestingly, the CdaI protein is not detectable in non-dividing cells, but in early stages of cell division localizes at the ciliary rows of the anterior half of the cell. As division progresses, the protein accumulates, concomitant with Mob1, at the region where the future furrow will be determined, and the future posterior pole of the anterior daughter cell will constitute (Tavares et al., 2012; Jiang et al., 2017). Depletion of CdaI from cells causes the division plane to displace anteriorly (Jiang et al., 2017). Interestingly, the posterior boundary of the CdaI labeled region, corresponding to the half anterior region of the cell, is affected by the *Elo1* gene. This suggests the existence of an interplay between the CdaI and the ELO1 proteins in the definition of the size of this region (Jiang et al., 2017, 2019). Interestingly, the previously described mutation in the *Elo1* gene, *elo1-1*, causes the shift of the oral primordium and the division plane toward the posterior pole of the cell (Frankel, 2008). The identity of the *Elo1* gene was determined by showing that it encodes an ortholog of the downstream NDR/Lats kinases of the Hippo pathway (Jiang et al., 2019). Similarly to Mob1, the Elo1 protein localizes at the posterior BBs in interphase, and is recruited to the midline in dividing cells (Tavares et al., 2012; Jiang et al., 2019). Additionally, loss-of-function of *Elo1* gene (Jiang et al., 2019) and depletion of CdaI are antagonistic, since the first moves the division plane to the posterior pole, whereas the second, moves to the anterior pole (Jiang et al., 2019). The overexpression of Elo1 phenocopies the loss of CdaI (Jiang et al., 2019). The double mutant *elo1-1_cdaI* starts to reveal the phenotype of the single *elo1-1* mutant but the phenotype progressively disappears probably due to compensation due to cdaI loss-of-function. Thus, Elo1 specifies the initial position of the division plane and CdaI maintains its equatorial localization and promotes nuclear divisions and cytokinesis (Jiang et al., 2019). Based on these observations the authors proposed that Elo1 and CdaI proteins operate in two consecutive Hippo signaling circuits sharing the same Mob1 (Figure 6) (Jiang et al., 2019).

It is possible that ciliates have different Hippo signaling routes using, for example, different kinases belonging to MST1/2 and NDR/LATS kinase families, or even different Mob molecules (accordingly to Jiang et al., 2019, *Tetrahymena* seems to have a Mob 4 homolog), but some pieces are still missing in this puzzle. It is now assumed that Mob1 transitorily assembles a unique complex containing both MST1/2 (upstream kinases) and NDR/LATS kinases (downstream kinases) to permit the activation of the LATS/NDR kinases by the MST1/2 kinases through a trans-phosphorylation event (Xiong et al., 2017). Moreover, this scenario is more complex because there are evidences that Mob1 can also interact, in a phosphorylation-dependent manner, with other signaling complexes, i.e., one complex containing the serine/threonine phosphatase PP6 (Couzens et al., 2013) and the Rho guanine exchange factors (DOCK6-8) (Mou et al., 2012; Xiong et al., 2017). Noticeable is the fact that the guanine nucleotide exchanger Dock6 is simultaneously specific for the signaling of protein Rac1 and for the rho-type GTPase Cdc42. The GTPase Cdc42 is an essential conserved factor in controlling eukaryotic cell polarity that its encoded by a gene present in the genome of *Tetrahymena* (Miyamoto et al., 2007; Kaczanowska et al., 2008). Although it is uncertain if these complexes participate in Hippo signaling pathway, this shows that Mob1 regulation and activity is much more complex than previously predicted.

A complete picture of the Mob1 role and the Hippo signaling pathway in ciliates is still missing but there is no doubt that different components of this signaling network present a polarized localization specifically throughout the antero-posterior axis. The mechanisms underlying this polarized distribution have not yet been uncovered. However, there is the possibility that other kinases operate upstream of the Hippo pathway, for example creating a first wave of signaling required for the recruitment of the Hippo components to specific localizations. In *Tetrahymena* there are evidences that support the hypothesis that GSK3β (Glycogen synthase kinase 3s)-MARK/MAPs signaling pathway may be involved in the establishment of global cell polarity (Kaczanowska et al., 2008). Although *Tetrahymena* genome does not seem to encode the components of a Wnt signaling pathway, that operate upstream of this signaling, the orthologs of the GSK3β-MARK2/PAR-1 pathway were identified (Kaczanowska et al., 2008). The MARK2/PAR-1 kinase is involved in the control of cell polarity by regulating the asymmetric localization of factors in a wide variety of polarized cells. For example, PAR-1 is critical for the first asymmetric division of the *C. elegans* zygote, for the establishment of the antero-posterior axis in the *Drosophila* oocyte and in the control of MT dynamics during neuronal morphogenesis (for review Wu and Griffin, 2017). The GSK3β kinase phosphorylates MARK2/PAR-1 kinase regulating its activity. One link between this cascade and the Hippo pathway appears to occur in metazoans through the protein disks large homolog 5 (DLG5) that plays a role in cell polarity and regulates cellular proliferation and differentiation (Kwan et al., 2016). DLG5 forms oligomeric complexes between the Hippo MST1/2 kinases and PAR-1 kinases and is a negative regulator of the Hippo signaling (Kwan et al., 2016). More recently, it was showed that the Tumor suppressor WWOX binds and blocks the function of GSK-3β kinase, also binding to upstream members of Hippo pathway with uncertain consequences (Chen et al., 2019). Both examples help to foresee that in *Tetrahymena*, like in metazoans, the interplay between members of the GSK3β-MARK2/PAR-1 signaling and the Hippo cascade may have important roles in morphogenesis and pattern perpetuation.

Based on the phenotypes of several mutants (Jerka-Dziadosz and Beisson, 1990; Frankel, 2008) it is conceivable that the components of the epiplasm and the infraciliary lattice in *Parmecium* may play a role in the crosstalk with distinct signaling cascades or components of these cascades during morphogenesis. For example the already mentioned knockdown of alveolin ALV2, that probably affects the cell’s epiplasm integrity, causes phenotypes that remind those of Mob1 (El-Haddad et al., 2013).

Ion gradients, like Ca^2+^, may also originate morphogenetic waves (Le Guyader and Hyver, 1991). Two calcium-binding proteins, TCBP-23 and TCBP-25 (Hanyu et al., 1995, 1996) are distributed throughout the epiplasm and the TCBP-23 seems to interact with the Epc1p. TCBP-23 depletion causes *Tetrahymena* abnormal cell shape, a similar phenotype to EPC1 knockout cells (Williams, 2004; Nakagawa et al., 2008). Also, centrins, that are calcium binding proteins, are good candidates to be sensors for Ca^2+^ gradients and/or transducers or effectors for Hippo pathway. Remarkably, in budding yeast centrin Cdc31 is required for cell integrity/morphogenesis, through the regulation of the Kic1p protein kinase (Sullivan et al., 1998). Noteworthy, Kic1p is a Hippo-like kinase, and a component of the Regulation of Ace2 and Morphogenesis (RAM) network, that operates during the M to G1 transition and cytokinesis where is required for the removal of the septum existing between the yeast mother and daughter cells (Weiss, 2012). In this signaling network Mob2 forms a complex with the NDR-related kinase Cbk1 that is activated by Kic1 (Nelson et al., 2003; Hsu and Weiss, 2013). These observations clearly demonstrate that centrins have important roles in morphogenesis by regulating Hippo-like kinases. According to the described phenotypes it is tempting to hypothesize that these proteins, by coordinating duplication, positioning and asymmetries of BBs, together with Hippo signaling help to define ciliate patterning.

## From Ciliates to Metazoans: a Journey of Discoveries

For more than six decades, work with ciliates focused on the comprehension of how their magnificent cortical pattern is hereditable, making critical contributions for the understanding of the mechanisms underlying cell polarity. These studies clearly indicated that there are micro-polarities, mainly centered in the structural and biochemical asymmetries of BBs that are explored by the inductive signals during cell division. Also, the maintenance of correct cell polarity is in close interplay with the accuracy of cell division, which is inevitably required to perpetuate morphology through this cellular process.

The patterning on a single cell begins with the establishment of a polarity axis, which resembles embryonic patterning that initiates with the polarization of the body axes. In fact, in several organisms like *C. elegans*, *Drosophila*, and *Xenopus*, the main body axis of the animal is defined by the polarity of the initial single-cell (Shulman and St Johnston, 1999; Castanon and González-Gaitán, 2011). This suggests that, the establishment of cell polarity is an ancient process, and the core mechanisms of cell polarization should be conserved throughout evolution. This idea has been supported by the studies in yeast that have shown that yeast polarity genes have similar functions in higher eukaryotes. Nevertheless, due to the vast and complex morphological patterns that cells can generate, yeast is likely a limited single-cell organism model for understanding such cell polarity mechanisms’ diversity. Moreover, yeast does not possess centrosomes/BBs, complex structures that in metazoans play central roles in cell polarity and morphogenesis. Centrosomes are critical for these events not only by being involved in spindle positioning, cytoskeleton organization and cilia assembly, but also by active participation in signaling cascades. For example, in *Drosophila*, the asymmetric cell division of male stem cells and neuroblasts determines which daughter cell will be maintained as a stem cell and which one initiates a differentiation program. The controlled orientation of the mitotic spindle seems to play a critical role in this event (Yamashita et al., 2007; Cheng et al., 2008; Yamashita and Fuller, 2008), which is linked to the intrinsic asymmetry of the centrioles of the centrosome (Rebollo et al., 2007; Cheng et al., 2008; Wang et al., 2009). The fate of the two daughter cells is connected to structural differences between the mother and daughter centrosomes (that contain the older and the youngest centrioles, respectively). These differences are related to the ability of the mother centrosome to assemble a primary cilium and organize more robust MTs asters, which allows the cell to be challenged by environmental signals (Yamashita and Fuller, 2008; Nigg and Raff, 2009). Therefore, it is conceivable that centrosomes may differentially harbor mechanisms and/or accumulate fate determinants that allow them to sense and interpret chemical/mechanical intrinsic and/or spatial extrinsic signals.

Ciliates offer incredible potentialities to uncover and explore these mechanisms. Indeed, *Tetrahymena* and *Paramecium* not only have huge number of BBs, probably the ancestors of centrosomes, but also possess different populations of BBs with distinct biochemical and structural composition. Moreover, this composition seems to be dynamic throughout the cell cycle and probably responds to environmental changes (Cole et al., 2008; Tavares et al., 2012; Jiang et al., 2017, 2019). For example, starved *Tetrahymena* cells are able to assemble new cilia from unciliated BBs, normally detected in preferential regions of the cortex (Nelsen and Debault, 1978). Some of these BBs localize in the posterior pole of the cell, a region where most of the BBs do not undergo duplication during cell division and are differently inherited by the two siblings (Figure 5). Furthermore, the posterior region is enriched in old BBs and corresponds to the region where Mob1 and Elo1 accumulate and is only inherited by the posterior daughter cell (Tavares et al., 2012; Jiang et al., 2019). These regions designated as invariant regions are also present in *Paramecium* (see Figure 5). In animal cells, mother and daughter centrioles are distinguishable by the presence of a set of distal and subdistal appendages at the mother centriole that are indispensable for anchoring the MTs and the centriole to the membrane during its transformation into a BB. Beside the appendage proteins, other proteins contribute for the identity of the mother centriole. For example, the polarity protein partitioning defective 6 homolog gamma (Par6c) is a component of the mother centriole that is important for the regulation of centrosomal protein composition and consequently for ciliogenesis, MT organization and centrosome reorientation during migration (Dormoy et al., 2013). In human cells, Mob1 supposedly concentrates in the mother centriole during late telophase (Florindo et al., 2012). Thus, it is tempting to make a parallel between the implications of the mother centrosome in human cells in the maintenance of the identity and spatial localization of stem and progenitor cells, and the implication of *Tetrahymena* old posterior BBs in the maintenance of cell patterning.

**FIGURE 7:**
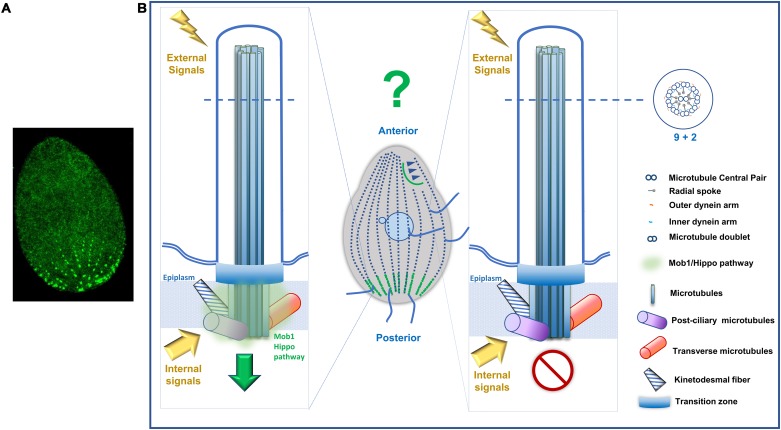
*Tetrahymena* Mob1 accumulates in distinct populations of basal bodies. **(A)** Immunofluorescence microscopy of a *T. thermophila* exponentially growing cell expressing Mob-GFP that concentrates in posterior region basal bodies. **(B)** Hypothetical model. Distinct basal bodies and corresponding assembled cilia enriched in Mob1/Hippo pathway components may differentially allow the concentration of intrinsic and/or extrinsic signals. This will create territories with different biochemical features that locally regulate specific effectors.

Motile cilia are generally regarded as machines able to generate movement but it is obvious that they are also sensory organelles (Jennings, 1899; Bloodgood, 2010). Although this subject is poorly explored, the ability of *Tetrahymena* to change cilia number and morphology in response to nutrient scarcity is a clear illustrative example. The Hippo pathway was firstly correlated with cilia in *Tetrahymena* upon the observation that Mob1-gene was upregulated in response to ciliogenesis, with cells depleted in Mob1 showing a delay in cilia recovery after deciliation (Tavares et al., 2012). More recently, important proteins involved in ciliogenesis, like EXOC5, were shown to regulate the Hippo pathway through Mob. Depletion of EXOC5 causes increased phosphorylation of Mob and loss of cilia (Lobo et al., 2017). Furthermore, MST1/2 kinase is required for phosphorylation of Aurora kinase A preventing its interaction with the HDAC6 desacetylase to avoid primary cilia disassembly. Additionally, MST1/2-SAV1 promotes ciliogenesis via association with the NPHP complex that controls the cargoes loading into intraflagellar transport (IFT) complexes at the cilium transition zone (Kim et al., 2014). The NPHP proteins constitute a hot spot in ciliopathies since they are, for example, mutated in patients with nephronophthisis. There are now several evidences that NPHP proteins regulate the Hippo pathway through the interaction with different components and promoting phosphorylation and nuclear translocation of pathway activators (for review Wheway et al., 2018). Perhaps, in ciliates the existence of populations of BBs enriched in Hippo pathway components allows the concentration of intrinsic and/or extrinsic signals in specific BB territories that consequently create different biochemical features to locally regulate specific effectors (Figure 7).

It has been argued that ciliates are not good models to study metazoan ciliated tissues and polarity. Although they have been contributing through the years to a better understanding of cilia biogenesis and structure/function, and to perceive the polarity mechanisms operating in a single cell, they appear as limited models to understand the polarity of multicellular ciliated epithelia. We can approach this problem in a positive way. In a simplistic view, the acquisition of multicellularity and consequently the emergence of different cell types and tissues required that cells integrate signaling and regulatory cascades in new environments including cell-cell and cell-matrix connections. A dramatic difference between single cell and multicellular organisms is the fact that most of the cells within multicellular entities do not directly respond to external environmental changes but, instead, receive information from this environment essentially by receptors in the cell membrane from the organism internal signals. This causes the addition of new layers of regulatory mechanisms and an increased complexity of signaling pathways. However, most probably, the core modules of these signaling cascades are conserved. Therefore, single cells organisms can easily reveal us these core mechanisms and how they operate inside a single complex cell. They are also good models to highlight regulatory steps that are probably diluted in the complex regulation of cells that live constrained by multiple cell–cell interactions and specific extracellular matrix environments. Illustrating this idea, the Yorkie/YAP components of the Hippo pathway are repressed by the transmembrane protein Crumbs, a key apical domain determinant, that is localized in the developing wing of *Drosophila* and a component of the planar cell polarity signaling pathway (PCP) that refers to the polarity of a cell within the plane of an epithelium (Chen et al., 2010; Ling et al., 2010; Grzeschik et al., 2010; Robinson et al., 2010). This shows that Hippo pathway is now operating in multicellular organism’s polarity in close relationship with the general polarity of tissues. Additionally, the family of transcriptional co-activators YAP/TAZ has been proposed to act as polarity sensors, mechano-sensors and damage-sensors *in vivo* (for review Elbediwy et al., 2016).

In metazoans the possibility that the maintenance of morphology also relies on self-organization has not been given much attention. Studies in ciliates clearly demonstrate the importance of self-organization, local polarities and spatial information. It has been consistently shown that the BB associated structures (the post ciliary, the transverse MTs and the rootlet) play an important role in defining positional information that will also be important for internal cell organization. These roles probably go beyond their accepted role as support/stabilizing structures for BBs and motile cilia. For example, in *Drosophila*, the component of rootlets, Rootletin (Root), is not required for cilia structure but its absence affects BB cohesion and the ciliary rootlet organization, compromising the sensory function of neurons. Mutant flies lacking rootlets showed mechano-sensation and chemo-sensation behavioral defects (Chen et al., 2015).

In multi-ciliated cells of epithelia tissues multiple centrioles arise via two independent routes. The centriole-dependent pathway (which contributes to 5% of centriole population in ciliated cells) and the deuterosome-dependent pathway also designated *de novo* pathway (responsible for 95% of the centriole population in ciliated cells) (for review Shahid and Singh, 2018). Although cortical BBs of ciliates arise by a centriole-dependent pathway, the ability to assemble BBs *de novo* is also present in those that can go through encystment. During excystment the new BBs are in fact assembled without the presence of any mature BB (Hanson, 1962; Jerka-Dziadosz, 1964; Grimes, 1973; Hammersmith, 1976; Hammersmith and Grimes, 1981), probably by mechanisms similar to those operating in the metazoans *de novo* pathway. Additionally, it was proposed that the OA BBs may arise from *de novo* assembly, and their orientation from the “anarchic field” is equivalent to the process of BB orientation in vertebrate multi-ciliated cells (for review Bayless et al., 2016). On the other hand, it is now well established that in ciliopathies many of the phenotypes may be due either to mutations in a single gene (Reiter and Leroux, 2017) or to mutations occurring in a number of different loci but originating similar phenotypes and causing the same disease. The scenario is even more complex since many gene mutations that lead to ciliopathies occur in genes that encode cilia core components but produce tissue specific phenotypes. These evidences support the idea that although cilia structure/function has been conserved throughout evolution a certain degree of variability is present. Moreover, specific proteomes and probably cilia-type specific functions for core components may also exist. In fact, a recent study conducted in *Drosophila melanogaster* shows that mutations in core cilia components can produce some of the human ciliopathies complex tissue-specific phenotypes as a result of ultra-structural variations of ciliary associated structures, namely, their specific protein composition and localization (Jana et al., 2018). The range of variability of cilia structures and its impact in function in metazoan tissues is still not well documented. What make ciliates unique is the fact they present cilia structural/functional diversity in a single cell and simultaneously the ability to adjust them in response to variations in their environment. Together, the discussed examples show that ciliates are excellent models for multi-ciliated epithelia.

## Conclusion and Future Perspectives

In conclusion, ciliates offer unforeseen opportunities to study the role of BBs as sensory platforms, distinct cilia in a single cell, and complex microtubular structures and their role “as relays in transmission of polarities and morphogenetic information” (quoted in Beisson and Jerka-Dziadosz, 1999). This is also true for the core mechanisms underlying polarity and morphogenesis that are still present in multicellular organisms. Although ciliates have massively contributed to the identification and functional understanding of tubulin/MT post-translational modifications, the way in which these modifications constitute an additional layer of information for perpetuation of ciliate cortical pattern during division is far from being exhausted. In this field, ciliates could still give an indispensable contribution to understand metazoan development.

Over time, ciliates not only gave new information about numerous basic processes of eukaryote cells but also have challenged the scientific community by leading to the formulation of new concepts. In this scenario, the compiled information concerning the role of the specific subpellicular layer of these organisms, indicating the existence of filamentous meshworks as guiding scaffolds during the morphogenesis, asks for attention. To understand how these proteins were replaced/evolved, and which of the actual proteins in higher eukaryote cells still play similar roles, will most probably contribute to new information of metazoan cortex as an interface for relationships with neighbor cells and the extracellular matrix. We envisage that this will impact in the rapid growing field of mechanobiology. To approach this we foresee that Atomic Force Microscopy (AFM) (Binnig et al., 1986) will be a strong tool. This is a powerful high-resolution microscopy technique, able to image surfaces and determine structural and mechanical properties of a variety of biological materials with the ability to provide a spatial resolution in the order of the nanometers in three dimensions without the need of vacuum or contrast reagents (Cai, 2018; Stylianou et al., 2019). Taken together all the capabilities of AFM, the potential for its use in ciliates polarity is huge. Although few studies have been made so far using AFM in the ciliate’s field, the ones that were made were successful. As an example, Seixas and colleagues were able to explore the potentialities of the technique to propose for the first time a model that describes the assembly of the cilia cap structure (Seixas et al., 2017). With the recent advances of high-speed AFM, tip functionalization and super resolution microscopy coupling the utility of AFM in ciliates studies is even bigger. Using molecular mapping AFM can help to understand cilia membrane composition dynamics and variances in the different regions of the cell at the nanometer range; with the correlation with STED or STORM we can better understand the biochemical polarities that underlie cortical structural polarities. All in all, AFM and ciliates have a high potential to deliver outstanding scientific findings.

## Author Contributions

HS defined the structure and wrote most of the review. BC and LV wrote part of the review more specifically focused on AFM. SN wrote part of the review and created the figures and legends.

## Conflict of Interest

The authors declare that the research was conducted in the absence of any commercial or financial relationships that could be construed as a potential conflict of interest.
